# Nontypeable Haemophilus influenzae Responds to Virus-Infected Cells with a Significant Increase in Type IV Pilus Expression

**DOI:** 10.1128/mSphere.00384-20

**Published:** 2020-05-27

**Authors:** Elaine M. Mokrzan, Kolapo A. Dairo, Laura A. Novotny, Lauren O. Bakaletz

**Affiliations:** a Center for Microbial Pathogenesis, Abigail Wexner Research Institute at Nationwide Children’s Hospital, Columbus, Ohio, USA; b Department of Pediatrics, The Ohio State University College of Medicine, Columbus, Ohio, USA; National Institute of Allergy and Infectious Diseases

**Keywords:** COPD, PilA, vaccine, adenovirus, otitis media, respiratory syncytial virus, rhinovirus

## Abstract

Nontypeable Haemophilus influenzae (NTHI) colonizes the human nasopharynx, but when the host immune response is dysregulated by upper respiratory tract (URT) virus infection, NTHI can gain access to more distal airway sites and cause disease. The NTHI type IV pilus (T4P) facilitates adherence, benign colonization, and infection, and its majority subunit PilA is in clinical trials as a vaccinogen. To further validate the strategy of immunization with PilA against multiple NTHI-induced diseases, it is important to demonstrate T4P expression under microenvironmental conditions that predispose to NTHI infection of the airway. Because URT infection commonly facilitates NTHI-induced diseases, we examined the influence of ongoing virus infection of respiratory tract epithelial cells on NTHI T4P expression *in vitro*. Polarized primary human airway epithelial cells (HAEs) were sequentially inoculated with one of three common URT viruses, followed by NTHI. Use of a reporter construct revealed that NTHI upregulated *pilA* promoter activity when cultured with HAEs infected with adenovirus (AV), respiratory syncytial virus (RSV), or rhinovirus (RV) versus that in mock-infected HAEs. Consistent with these results, *pilA* expression and relative PilA/pilin abundance, as assessed by quantitative reverse transcription-PCR (qRT-PCR) and immunoblot, respectively, were also significantly increased when NTHI was cultured with virus-infected HAEs. Collectively, our data strongly suggest that under conditions of URT virus infection, PilA vaccinogen induction of T4P-directed antibodies is likely to be highly effective against multiple NTHI-induced diseases by interfering with T4P-mediated adherence. We hypothesize that this outcome could thereby limit or prevent the increased load of NTHI in the nasopharynx that characteristically precedes these coinfections.

**IMPORTANCE** Nontypeable Haemophilus influenzae (NTHI) is the predominant bacterial causative agent of many chronic and recurrent diseases of the upper and lower respiratory tracts. NTHI-induced chronic rhinosinusitis, otitis media, and exacerbations of cystic fibrosis and chronic obstructive pulmonary disease often develop during or just after an upper respiratory tract viral infection. We have developed a vaccine candidate immunogen for NTHI-induced diseases that targets the majority subunit (PilA) of the type IV twitching pilus (T4P), which NTHI uses to adhere to respiratory tract epithelial cells and that also plays a role in disease. Here, we showed that NTHI cocultured with virus-infected respiratory tract epithelial cells express significantly more of the vaccine-targeted T4P than NTHI that encounters mock-infected (healthy) cells. These results strongly suggest that a vaccine strategy that targets the NTHI T4P will be effective under the most common predisposing condition: when the human host has a respiratory tract virus infection.

## Abstract (precis)

Nontypeable Haemophilus influenzae (NTHI) is the predominant bacterial causative agent of many chronic and recurrent diseases of the upper and lower respiratory tracts. NTHI-induced chronic rhinosinusitis, otitis media, and exacerbations of cystic fibrosis and chronic obstructive pulmonary disease often develop during or just after an upper respiratory tract viral infection. We have developed a vaccine candidate immunogen for NTHI-induced diseases that targets the majority subunit (PilA) of the type IV twitching pilus (T4P), which NTHI uses to adhere to respiratory tract epithelial cells and that also plays a role in disease. Here, we showed that NTHI cocultured with virus-infected respiratory tract epithelial cells express significantly more of the vaccine-targeted T4P than NTHI that encounters mock-infected (healthy) cells. These results strongly suggest that a vaccine strategy that targets the NTHI T4P will be effective under the most common predisposing condition: when the human host has a respiratory tract virus infection.

## INTRODUCTION

Nontypeable Haemophilus influenzae (NTHI) is a pathobiont of the human nasopharynx. Prior or concurrent upper respiratory tract virus infection dysregulates host airway epithelial defenses (1) and increases the expression of proteins that NTHI subsequently uses to adhere to and colonize the respiratory tract epithelium (2–5). In the nasopharynx, this increased colonization load allows NTHI to gain access to more distal sites of the airway, where it causes disease (6–8). During NTHI-induced otitis media (OM), rhinosinusitis, and exacerbations of cystic fibrosis and chronic obstructive pulmonary disease (COPD), NTHI forms biofilms at the site of infection that are highly resistant to killing by either the host immune system or by antibiotics (9–11) and thereby contribute significantly to the chronic and recurrent nature of these infections (12–15). Thus, novel strategies to prevent and/or treat NTHI-induced diseases are needed.

The type IV pilus (T4P) of NTHI is important for adherence to and colonization of respiratory tract epithelial cells, twitching motility, biofilm formation, and competence (16–20). Antibodies against the majority subunit of the T4P, PilA, prevent the formation of and/or disrupt existing NTHI biofilms *in vitro* (17, 21, 22). Furthermore, antibodies against PilA prevent the development of, as well as therapeutically resolve, existing experimental NTHI-induced OM in chinchilla models (21, 23–25). Due to the importance of T4P for NTHI colonization and pathogenesis, and as a result of the conservation of the amino acid sequence of PilA among diverse NTHI strains (16, 26, 27), PilA is in clinical trials as a candidate vaccine immunogen for the prevention of NTHI-induced exacerbations of COPD (28, 29). To further validate the strategy of immunization with PilA against multiple NTHI-induced diseases, it is important to demonstrate T4P expression under microenvironmental conditions that commonly predispose to these diseases and, specifically, under conditions of viral coinfection.

Many respiratory tract infections are polymicrobial (30–33), and viral upper respiratory tract (URT) infection frequently precedes bacterial coinfection (34–36). URT virus infection induces many alterations in the host epithelium, some of which favor bacterial adherence and colonization (37). These changes include decreased ciliary beat frequency (38–40), decreased expression of antimicrobial peptides (41, 42), dysregulation of nutritional immunity (43, 44), and upregulated expression of eukaryotic cell surface proteins that become ligands for bacterial adhesins (2–5, 34, 45). In the presence of healthy human airway epithelial cells, NTHI upregulates T4P expression even prior to contacting, or adhering to, these cells (46). However, the effects of prior URT virus infection of respiratory tract epithelial cells on subsequent expression of T4P by NTHI have not been studied. Accordingly, here, we examined T4P expression by NTHI cultured with well-differentiated primary human airway epithelial cells (HAEs) with ongoing infection due to adenovirus (AV), respiratory syncytial virus (RSV), or rhinovirus (RV), three of the most common URT viruses associated with NTHI coinfections (38, 47–52).

## RESULTS

**FIG 1:**
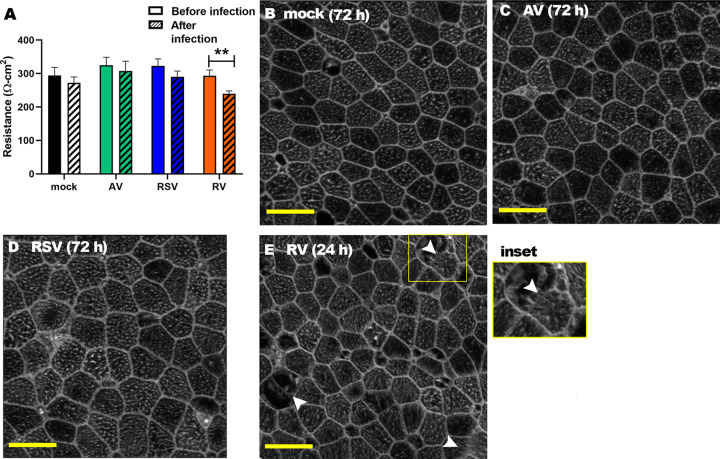
Assessment of HAE culture integrity after virus infection. (A) TEER of HAE cultures was measured before and after mock, AV, RSV, or RV infection. As anticipated, only infection with RV caused a significant decrease in TEER. **, *P* < 0.01 (B to E) Microscopy images of the apical surfaces of HAE cultures stained for F-actin to visualize cellular junctions. Clear, uninterrupted cell-cell junctions were evident for mock- (B), AV- (C), or RSV-infected (D) HAEs. (E) RV-infected HAEs showed localized areas of disrupted epithelial cell junctions (indicated by white arrows and shown in inset) but no loss of cells. Together, these data confirmed that HAEs could serve as a relevant *in vitro* model of virus-infected human airway epithelium. Bars, 10 μm.

To examine the influence of ongoing URT virus infection of the human airway on NTHI T4P expression, we utilized HAEs grown at the air-liquid interface as a surrogate *in vitro* model. In this culture system, HAEs form a well-differentiated pseudostratified epithelium that recapitulates the human airway epithelium in both appearance and function (53, 54). To first confirm that virus infection at the multiplicity of infection (MOI) used herein did not destroy the integrity of the epithelial barrier, we inoculated HAEs with AV, RSV, or RV as previously described (2, 55–58) and monitored transepithelial electrical resistance (TEER) as a measure of epithelial barrier integrity. We observed minimal changes in TEER after mock infection or infection with AV or RSV (Fig. 1A), as anticipated (55, 56), and light micrographs of the apical surface showed discrete F-actin labeling that clearly defined the cell-cell junctions of mock-, AV-, or RSV-infected HAEs (Fig. 1B to D, respectively). Unlike AV or RSV, however, RV is cytolytic and disrupts cell-cell junctions (57, 58); thus, a significant drop in TEER values of RV-infected HAEs was both expected and observed (Fig. 1A). Light microscopy revealed a few localized areas of disrupted cell-cell junctions, but there was no loss of cells (Fig. 1E). Thereby, for all subsequent experiments, we infected HAEs with either RSV or AV for 72 h, but only for 24 h with RV. Taken together, these data confirmed that HAEs could serve as a relevant *in vitro* model of confluent virus-infected human airway epithelium with which to measure their influence on T4P expression by NTHI.

**FIG 2:**
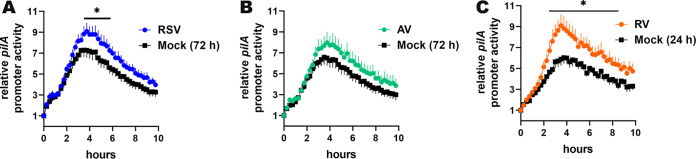
NTHI *pilA* promoter activity was stimulated by coculture with virus-infected HAEs. Mock- or virus-infected HAEs were inoculated with NTHI 86-028NP/pKMLN-02, and luminescence was monitored to estimate relative *pilA* promoter activity. (A) Prior RSV infection induced significantly greater NTHI *pilA* promoter activity than in cultures with mock-infected HAEs. (B) A similar trend was observed for AV-infected HAEs. (C) Coculture with RV-infected HAEs induced a sustained significant increase in *pilA* promoter activity versus that in mock-infected HAEs. These data suggested that *pilA* promoter activity was stimulated when NTHI was cocultured with RSV-, AV-, or RV-infected HAEs. *, *P* < 0.05 versus mock infected.

To estimate relative NTHI T4P expression during coculture with URT virus-infected HAEs, we used an NTHI reporter in which luciferase expression is driven by the *pilA* promoter and monitored luminescence over time as a surrogate for T4P expression (18). For NTHI cocultured with mock-infected HAEs, relative *pilA* reporter activity increased over 4 h and then decreased slowly thereafter (Fig. 2A to C, black lines). In comparison, *pilA* reporter activity was further stimulated when NTHI was cocultured with HAEs that were previously infected with any of the three URT viruses tested (Fig. 2A to C, colored lines). Promoter activity increased within 3.5 h and was significantly so from 3.5 to 5.75 h of NTHI coculture with RSV-infected HAEs (Fig. 2A) (*P* ≤ 0.05 versus mock infected). We observed a similar increase in promoter activity for NTHI cultured with AV-infected HAEs that began at 2.75 h, although the difference from mock-infected HAEs was not statistically significant (Fig. 2B). NTHI cultured with RV-infected HAEs significantly increased relative *pilA* promoter activity from 2.5 to 8.5 h of coculture (Fig. 2C) (*P* ≤ 0.05). Moreover, even though the duration and magnitude of the promoter activity increase was unique for each URT virus tested, in each case, relative promoter activity remained greater for NTHI cultured with virus-infected HAEs for at least 10 h of coculture (Fig. 2A to C). Together, these data showed that *pilA* promoter activity was significantly greater when NTHI was cocultured with virus-infected than with mock-infected (e.g., “healthy”) HAEs.

Our *pilA* promoter activity results suggested that NTHI expressed more PilA/T4P when cultured with virus-infected HAEs. However, prior virus infection of URT epithelial cells can also stimulate bacterial growth, due to dysregulated nutritional immunity (43, 44). Accordingly, we quantified NTHI CFU after coculture. As anticipated, ongoing HAE infection with each of the URT viruses tested did indeed stimulate NTHI growth over the 10 h of the incubation period (see Fig. S1 in the supplemental material) (*P* < 0.05 versus mock infected at 10 h). Since this enhanced growth could have affected our estimates of *pilA* promoter activity and, by inference, T4P expression, we next examined the effect of prior URT virus infection via assessment of relative *pilA* transcript abundance normalized to 16S rRNA.

10.1128/mSphere.00384-20.1 FIG S1 NTHI growth was stimulated by coculture with virus-infected HAEs. After 10 h, there were significantly more CFU of NTHI in cocultures with AV-, RSV-, or RV-infected than with mock-infected HAEs. *P < 0.05; **P < 0.01 versus mock infected at 10 h. Download FIG S1, TIF file, 0.1 MB. Copyright © 2020 Mokrzan et al. 2020 Mokrzan et al. This content is distributed under the terms of the Creative Commons Attribution 4.0 International license.

**FIG 3:**
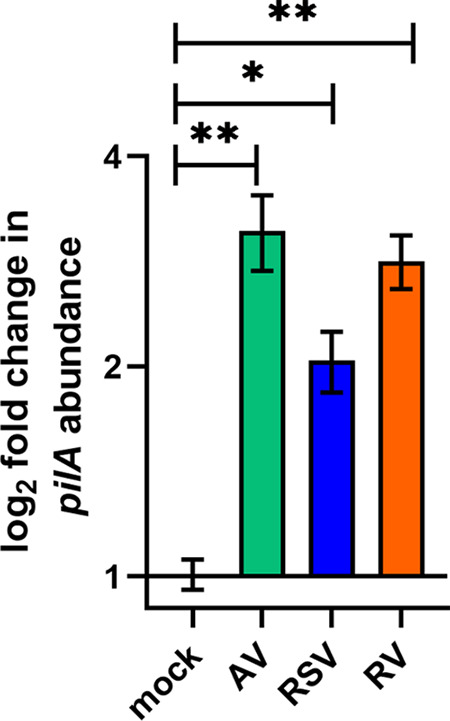
NTHI *pilA* expression was upregulated during coculture with virus- versus that in mock-infected HAEs. NTHI were cocultured with mock-infected or virus-infected HAEs. After 3 h, planktonic NTHI was collected and relative expression of *pilA* was assessed by qRT-PCR. NTHI cocultured with AV-, RSV-, or RV-infected HAEs displayed significantly greater *pilA* expression than NTHI cultured with mock-infected HAEs. *, *P* < 0.05; **, *P* < 0.001.

With the knowledge that NTHI upregulates *pilA* expression prior to contact with, or adherence to, healthy human airway epithelial cells (46), we isolated RNA from planktonic NTHI after coculture with virus- or mock-infected HAEs and used quantitative reverse transcription-PCR (qRT-PCR) to measure relative *pilA* transcript abundance. After 30 min, relative *pilA* expression was similar for all culture conditions (data not shown). However, after 3 h of coculture, NTHI cultured with AV-, RSV-, or RV-infected HAEs expressed significantly more *pilA* (Fig. 3) (*P *≤ 0.05 versus mock infected). These results correlated well with *pilA* promoter activity (Fig. 2) and, together, suggested that NTHI which encounters a URT virus-infected HAE would increase T4P expression above that of NTHI that encounters a healthy HAE.

**FIG 4:**
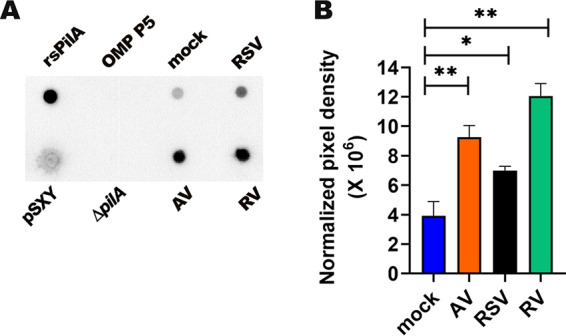
PilA/pilin protein was more abundant in lysates of NTHI incubated for 4 h with virus-infected than with mock-infected PBTEs. NTHI was cocultured with PBTEs for 4 h and probed with anti-rsPilA IgG. (A) Representative immunoblot showed strong reactivity with rsPilA and lysate of NTHI *rpsL*(pSXY), which overexpresses T4P, but not with isolated OMP P5 or lysates of NTHI Δ*pilA*. There was greater reactivity with lysates of NTHI cocultured with RSV-, RV-, or AV-infected PBTEs than with mock-infected PBTEs (compare the 4 dots on the right half of blot). (B) Pixel intensities of dots for NTHI lysates from 3 separate blots were normalized to CFU NTHI per sample. Relative pixel density was significantly greater for lysates of NTHI cocultured with AV-, RSV-, or RV-infected than with mock-infected PBTEs. These data showed significantly more PilA/pilin protein in lysates of NTHI cocultured for 4 h with virus-infected than with mock-infected PBTE cells. *, *P* < 0.05; **, *P* < 0.01 versus mock infected.

To further complement the evidence that supported upregulation of T4P by NTHI, we used an immunoblot assay to estimate the relative abundance of PilA and/or pilin protein in whole-cell lysates of planktonic NTHI recovered after coculture with mock- or virus-infected primary bronchial/tracheal epithelial cells (PBTEs). As expected, rabbit polyclonal anti-recombinant soluble PilA (rsPilA) IgG was strongly reactive with both positive controls: rsPilA and lysate of an NTHI variant that overexpresses T4P (19) (Fig. 4A and S2A). Moreover, there was minimal reactivity with both negative controls: an unrelated NTHI outer membrane protein (OMP P5) and lysate of NTHI 86-028NP Δ*pilA*, which does not express T4P (19); results which collectively validated the specificity of the immunoblot assay for PilA and/or pilin proteins. As anticipated, there was no difference in PilA/pilin protein between lysates of NTHI cocultured with virus- or mock-infected PBTEs for 30 min, as could be expected since this time point preceded the increase in *pilA* transcription observed at 3 h (Fig. 3 and S2B). However, after 4 h of coincubation, lysates of NTHI cocultured with AV-, RSV-, or RV-infected PBTEs demonstrated significantly greater PilA/pilin protein abundance than NTHI from mock-infected PBTEs (Fig. 4A and B) (*P* ≤ 0.05). Collectively, our results for protein abundance, relative *pilA* expression, and *pilA* promoter activity were highly concordant and strongly suggested that NTHI upregulated T4P expression when cultured with, but prior to adherence to, HAEs that were previously infected with AV, RSV, or RV compared to that in mock-infected HAEs.

10.1128/mSphere.00384-20.2 FIG S2 PilA/pilin protein abundance was similar for lysates of NTHI incubated for 30 min with virus- versus mock-infected HAEs. NTHI were cocultured with PBTEs for 30 min and probed with anti-rsPilA IgG. (A) Representative immunoblot showed reactivity with rsPilA and lysate of NTHI *rpsL*(pSXY), which overexpresses T4P, but not with isolated OMP P5 or lysates of NTHI Δ*pilA*. There was similar reactivity amongst lysates of NTHI cocultured with RSV-, RV-, AV-, or mock-infected PBTEs. (B) Pixel intensities of dots for NTHI lysates from 3 separate blots were normalized to CFU NTHI per sample. These data showed that after 30 min, NTHI cocultured with virus- or mock-infected HAEs expressed similar amounts of PilA/pilin protein. Download FIG S2, TIF file, 0.1 MB. Copyright © 2020 Mokrzan et al. 2020 Mokrzan et al. This content is distributed under the terms of the Creative Commons Attribution 4.0 International license.

**FIG 5:**
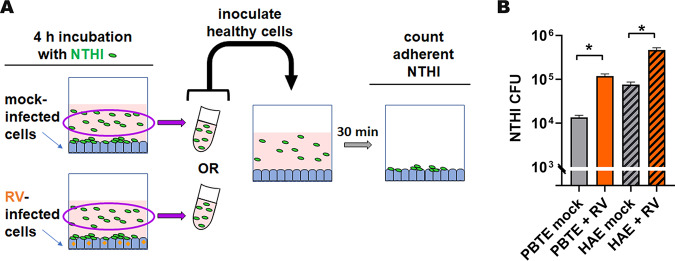
Prior coculture with RV- versus mock-infected epithelial cultures resulted in a subpopulation of NTHI with significantly increased ability to adhere to healthy PBTEs or HAEs. (A) Schematic for subculture adherence assay. NTHI was cocultured with either RV- or mock-infected PBTEs or HAEs for 4 h, after which, the planktonic NTHI above these virus-infected cells was collected and inoculated on healthy (uninfected) PBTE or HAE cultures. After 30 min, adherent NTHI were enumerated. (B) CFU were normalized to compensate for differences between the relative concentrations of NTHI recovered from RV-infected or mock-infected cocultures. After 30 min, NTHI collected from coculture with RV-infected PBTEs or HAEs was significantly more adherent to healthy PBTEs or HAEs, respectively, than NTHI collected from coculture with mock-infected cells. These results demonstrated that the upregulation of T4P expression after coculture with virus-infected epithelial cells significantly augmented the ability of NTHI to adhere. *, *P* < 0.05.

Due to the importance of T4P for NTHI adherence to respiratory tract epithelial cells and colonization of the nasopharynx, twitching motility, biofilm formation, and competence (16–20), we hypothesized that the increase in T4P expression reported here would likely have important biological consequences for NTHI. To test this hypothesis, we assessed relative adherence to respiratory tract epithelial cells by NTHI that had already been cocultured with RV-infected cells compared to those that had already been cocultured with mock-infected PBTEs or HAEs. After coculture for 4 h, we collected the planktonic NTHI and inoculated it directly onto healthy (uninfected) epithelial cell cultures (Fig. 5A). Planktonic NTHI collected from cocultures with RV-infected PBTEs or HAEs was significantly more adherent to healthy respiratory tract epithelial cells than NTHI collected from mock-infected cocultures (Fig. 5B) (*P* < 0.05). These results demonstrated that the upregulation of T4P expression induced by coculture with RV-infected PBTEs or HAEs did indeed promote adherence of NTHI to respiratory tract epithelial cells.

## DISCUSSION

NTHI T4P are essential for many important biological processes that include adherence, twitching motility, colonization, biofilm formation, and competence (16–20). Due to the importance of NTHI T4P during both asymptomatic colonization and infection, and because expression of the majority subunit of T4P, PilA, is highly conserved among diverse NTHI isolates (16, 26, 27), the vaccine candidate immunogen PilA is in clinical trials for protection against NTHI-induced exacerbations of COPD (28, 29).

To further validate PilA as a vaccinogen for use against multiple NTHI-induced diseases of the respiratory tract, T4P must be expressed during conditions encountered in the human airway prior to disease induction. Thereby, here we examined T4P expression specifically in the context of prior infection of respiratory tract epithelial cells with RSV, AV, or RV, as these viruses commonly predispose to NTHI disease (38, 47–52). Coinfection with RSV and NTHI is associated with bronchiolitis and subsequent wheezing in children (49), and RV infection facilitates the development of both rhinosinusitis (33) and NTHI-induced exacerbations of COPD (59, 60). Infection with RSV, AV, or RV is associated with increased risk of acute OM in children (32, 47, 61). Moreover, URT virus infection increases the rate of exacerbation due to NTHI in patients with cystic fibrosis (62).

NTHI upregulates T4P expression even before contacting or adhering to healthy human respiratory tract epithelial cells (46). However, NTHI-induced diseases commonly occur during, or immediately after, URT virus infection (34–36). Virus infection alters the environment of the respiratory tract by multiple mechanisms, which collectively facilitate overgrowth of NTHI in the nasopharynx and migration to more distal sites of the respiratory tract, where NTHI causes disease (6, 7, 41). The results of this study strongly suggest that NTHI expression of PilA/T4P will further increase when the host has a URT virus infection, due to the enhanced ability of NTHI to adhere to epithelial cells that line the respiratory tract and thereby contribute to an increased bacterial load in the nasopharynx. Thus, an immunization strategy that targets NTHI PilA/T4P will likely be highly effective against NTHI-induced diseases under the condition of virus coinfection, due to the increased expression of the vaccine target PilA and accessibility of the T4P on the surface of these “planktonic” (not yet adherent) bacterial cells. We hypothesize that under conditions of viral coinfection, anti-PilA antibodies will bind to NTHI that is expressing T4P and thereby block adherence, effectively limiting or preventing the highly characteristic and markedly increased bacterial load of NTHI that occurs in the human nasopharynx just prior to disease induction.

## MATERIALS AND METHODS

### NTHI variants and growth conditions.

NTHI variants used here are listed in Table 1. NTHI 86-028NP and NTHI 86-028NP Δ*pilA* were grown on chocolate agar at 37°C. For growth of NTHI 86-028NP/pKMLN-02, chocolate agar was supplemented with 20 μg kanamycin/ml to ensure retention of the plasmid, which contained a kanamycin resistance cassette.

**TABLE 1:** NTHI used in this study

Bacterial variant	Description	Reference
NTHI 86-028NP	Archived strain originally isolated from the nasopharynx of a child with chronic OM and maintained at low passage number	68
NTHI 86-028NP/pKMLN-02	NTHI 86-028NP reporter in which expression of luciferase is under control of the *pilA* promoter	18
NTHI 86-028NP *rpsL*(pSXY)	NTHI 86-028NP mutant engineered to overexpress T4P	19
NTHI 86-028NP Δ*pilA*	Nonpolar *pilA* mutant that does not express T4P	19

### Primary human airway epithelial cell culture and imaging.

Well-differentiated primary human airway epithelial cells (HAEs) of tracheobronchial origin, from two healthy donors, were obtained from the C3 Epithelial Cell Core at Nationwide Children’s Hospital. (Columbus, OH). Cells were seeded on 6.5-mm-diameter Transwell inserts (Corning Inc., Corning, NY) with a pore size of 0.4 μm. PneumaCult ALI medium (StemCell, Vancouver, BC) was added to the basolateral and apical compartments and changed 3 times per week. After 10 days, apical medium was permanently removed; however, feeding from the basolateral compartment continued with medium replaced 3 times weekly for ≥4 weeks prior to use to permit polarization and differentiation (63).

For immunofluorescence microscopy, HAEs were fixed overnight in 10% buffered formalin phosphate (Fisher) and stained with Alexa Fluor 594-labeled phalloidin (Invitrogen), per the manufacturer’s instructions. Cells were visualized via a Zeiss LSM 800 laser confocal scanning microscope (Zeiss) and images were rendered with Zeiss Zen software.

### Virus strains and infection of HAE cultures.

Respiratory syncytial virus strain A2 (RSV) and rhinovirus serotype 39 (RV) were purchased from American Type Culture Collection (Manassas, VA). Adenovirus serotype 1 (AV) is an archived pediatric clinical isolate (2, 38, 64, 65).

HAE cultures were inoculated with RSV, AV, or RV as previously described (2, 57). Briefly, the apical surface of each HAE culture was first rinsed with 10 mM Dulbecco’s phosphate-buffered saline (DPBS) to remove mucus and then inoculated with 50 μl of virus diluted in cell culture medium to a multiplicity of infection (MOI) of 0.1 for RV or 0.2 for RSV and AV, based on the number of epithelial cells exposed at the apical surface of the cultures. HAEs were incubated on a rocker at 34°C to mimic the temperature of the human nasopharynx (66, 67) for 2 h (RSV or AV) or 4 h (RV), as previously described (2, 57). The apical medium was then aspirated, and cultures were incubated for an additional 70 h for RSV or AV or 20 h for RV. Cultures inoculated with sterile cell culture medium served as negative controls. Transepithelial electrical resistance (TEER) was measured with an EVOM voltohmmeter (World Precision, Sarasota, FL) per the manufacturer’s instructions to assess the relative integrity of the HAE cultures before and after virus infection.

### Estimation of *pilA* promoter activity by NTHI cocultured with HAEs.

HAE cultures were mock or virus infected as described above and then rinsed with DPBS to remove mucus prior to apical inoculation with 50 μl of NTHI 86-028NP/pKMLN-02 to ∼3.5 × 10^7^ CFU/ml DPBS (MOI, 2:1). Plates were incubated in a FLUOstar Omega microplate reader with 5% CO_2_ at 34°C, and luminescence was measured every 15 min for 10 h. After subtraction of background, luminescence values of NTHI 86-028NP/pKMLN-02 were divided by their respective time zero readings. The ratios were plotted as relative fold change in *pilA* promoter activity. Data points represent the mean ± standard error of the mean (SEM) from 3 biological replicates, each measured in duplicate.

### Quantitation of NTHI.

NTHI 86-028NP was inoculated at an MOI of 2:1 on virus-infected or control HAE cultures as described above. After 10 h of incubation at 34°C, planktonic NTHI in coculture with HAEs was recovered from the apical chamber. To harvest the adherent NTHI, HAE cells were dissociated from the Transwell support by incubation with 200 μl TrypLE Select enzyme (Thermo Fisher Scientific, Waltham, MA) for 15 min at 37°C. We then combined the dissociated cells with adherent NTHI and the apical DPBS with planktonic NTHI from each Transwell and plated them on chocolate agar to enumerate CFU. Final values represent the mean ± SEM from 3 biological replicates, each measured in duplicate.

### Subculture adherence assay.

NTHI colonies collected from growth on chocolate agar were suspended in DPBS and inoculated on either RV-infected or control HAE cultures or on RV-infected or control PBTE cultures at an MOI of 0.1:1. After 4 h of incubation at 34°C, 50 μl of apical medium that contained planktonic NTHI was collected and inoculated on the apical surface of healthy (non-virus infected) HAEs or PBTEs (Fig. 5A). The second set of cultures were then incubated for 30 min at 34°C prior to harvest of cells and enumeration of adherent NTHI as described. It was noted that a greater number of planktonic NTHI were collected from RV-infected HAE or PBTE cultures than from mock-treated cultures (as per Fig. S1 in the supplemental material). Therefore, to permit direct comparisons between these groups we normalized the CFU collected from mock-infected PBTEs or HAEs as follows: [(CFU of NTHI collected from above RV-infected cocultures ÷ CFU of NTHI collected from above mock-infected cocultures) × CFU after 30 min incubation on second set of cells].

The adjusted CFU were compared by paired Student's *t* test. Three independent assays were performed, and the means ± SEMs are shown in the figures.

### RNA isolation and qRT-PCR analysis of *pilA* expression.

NTHI was inoculated on mock- or virus-infected HAE cultures as described above and incubated at 34°C. After 3 h, DPBS that contained planktonic NTHI was collected from the apical chamber, transferred into 1 ml TRIzol reagent (Thermo Fisher Scientific), and immediately stored at −80°C. RNA was purified with an RNeasy kit (Qiagen, Germantown, MD). Residual DNA was removed by treatment with DNase I (NEB, Ipswich, MA) for 45 min at 37°C in the presence of 20 U SUPERase In RNase inhibitor (Thermo Fisher Scientific). Relative expression of *pilA* was assessed by quantitative reverse transcription-PCR (qRT-PCR) with a TaqMan RNA-to-C_T_ 1-Step kit (Thermo Fisher Scientific), per the manufacturer’s protocol. Gene expression was normalized to 16S rRNA, and relative *pilA* expression calculated by the comparative threshold cycle (ΔΔ*C_T_*) method, with fold change in gene expression expressed as 2^−ΔΔ^*^CT^*. A fold change in gene expression of ≥2.0 was considered biologically relevant. Primers used for the TaqMan assay were designed by and can be obtained from Thermo Fisher Scientific via reference to the following identifiers (IDs): PILA (APAAAJN) for *pilA* and 16S (APDJXPH).

### Relative quantitation of PilA and/or pilin protein abundance by immunoblot.

The small surface area of the HAE cultures (0.33 cm^2^) limited our ability to collect enough planktonic NTHI to assay for PilA/pilin protein abundance. Instead, confluent monolayers of human primary bronchial/tracheal epithelial cells (PBTEs; ATCC, Manassas, VA) were established in T25 flasks and inoculated with PneumaCult EX Plus medium (StemCell Technologies) that contained RSV, AV, or RV as described above. After incubation on a rocker for 2 h (RSV and AV) or 4 h (RV), the culture medium that contained virus was replaced with sterile medium, and PBTEs were incubated for an additional 70 h for RSV or AV or 20 h for RV, which resulted in a total incubation time of 72 h (RSV and AV) or 24 h (RV). As a negative control, PBTEs were mock infected with culture medium alone for 72 h.

NTHI colonies were taken from chocolate agar, as this method of growth results in minimal T4P expression (16), suspended in DPBS, and inoculated on PBTE cells at an MOI of 2:1. After incubation at 34°C for 3 h, we collected the culture medium with its planktonic subpopulation of NTHI. A 20-μl aliquot was collected to determine CFU NTHI, and the remaining volume was centrifuged for 10 min at 20,800 × *g* in an Eppendorf 5430 microcentrifuge. The pellet was solubilized in B-PER reagent (Thermo Fisher Scientific) per the manufacturer’s instructions. Immunoblot analysis was performed as previously described (46). Solubilized samples were vacuum aspirated onto charged polyvinylidene difluoride (PVDF) LF membranes (Bio-Rad, Hercules, CA) in a Bio-Dot SF microfiltration apparatus (Bio-Rad). Membranes were blocked with DPBS plus 2% normal goat serum (Bethyl Laboratories, Montgomery, TX), 2% bovine serum albumin (Sigma-Aldrich, St. Louis, MO), and 0.05% Tween 20 (Fisher Scientific, Waltham, MA) for 1 h at 25°C and then incubated with 2.5 μg affinity-purified polyclonal rabbit anti-rsPilA IgG per ml DPBS overnight at 4°C. Membranes were washed and incubated with 1 μg goat anti-rabbit IgG conjugated to horseradish peroxidase (Molecular Probes, Eugene, OR) per ml DPBS for 1 h at 25°C. Blots were visualized by chemiluminescence with Clarity Western ECL substrate (Bio-Rad, Hercules, CA) per the manufacturer’s instructions and imaged with a FluorChem M system (ProteinSimple, San Jose, CA). To demonstrate positive reactivity of anti-rsPilA IgG, 0.01 μg purified rsPilA or a solubilized NTHI 86-028NP *rpsL*(pSXY), which overexpresses T4P, was used (19). NTHI 86-028NP outer membrane protein P5 (0.01 μg) and solubilized NTHI 86-028NP Δ*pilA*, which does not express T4P (19), served as negative controls. The density of each dot was measured with ImageJ 1.46r software (http://imagej.nih.gov/ij). Due to the potential for nonspecific increase in protein content as a result of the presence of PBTE cells, we normalized the pixel intensity value for each dot to CFU of NTHI (as determined by plate count) incorporated into that sample to directly compare the relative reactivity of anti-rsPilA IgG against each NTHI lysate. The assay was repeated 3 times on different days.

### Statistics.

All experiments were performed in duplicate with least 3 biological replicates. Data analyses were performed with GraphPad Prism software version 8.2.0. TEER measurements (Fig. 1A) and adherent NTHI (Fig. 5B) were compared by paired Student's *t* test. For multiple comparisons, one-way analysis of variance with the Holm-Šidák correction was used. All other comparisons were made with Student's *t* tests. Results are expressed as mean ± SEM. A *P* value of ≤0.05 was considered significant.
