# Supplementary figures and images for: Nontypeable Haemophilus influenzae Responds to Virus-Infected Cells with a Significant Increase in Type IV Pilus Expression
Source: mSphere. 2020 May 27;5(3):e00384-20. doi: 10.1128/mSphere.00384-20 (PMC7253600; doi:10.1128/mSphere.00384-20)

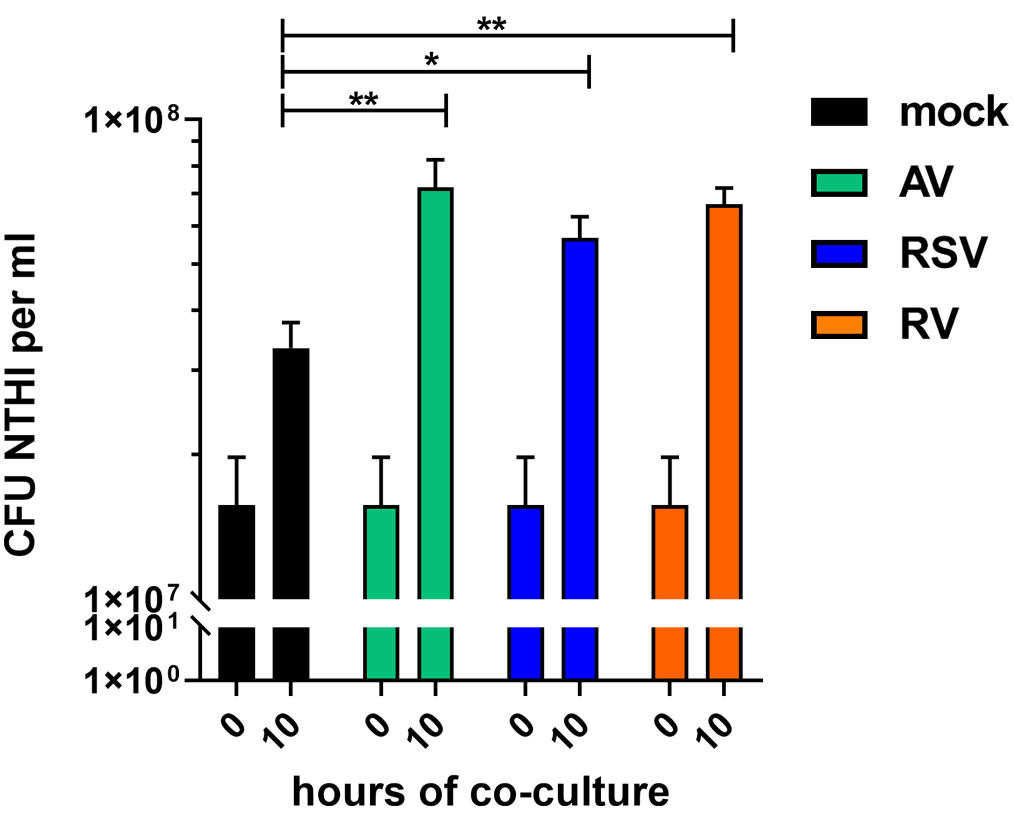

Supplement: FIG S1 [file mSphere.00384-20-sf001.tif]

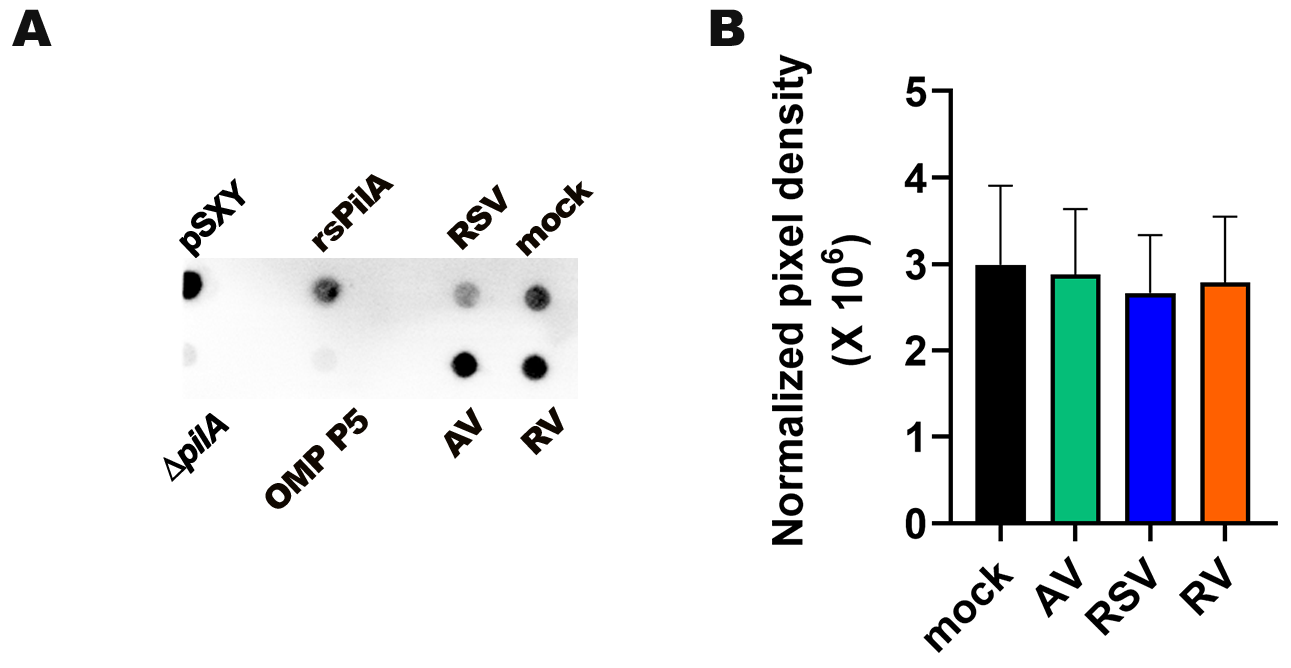

Supplement: FIG S2 [file mSphere.00384-20-sf002.tif]
